# Factors Influencing Uptake of Prostate Cancer Screening among Men Aged 40 Years and Above in Kazo Town Council, Kazo District, Uganda: A Cross-Sectional Study

**DOI:** 10.1155/2023/7770943

**Published:** 2023-12-26

**Authors:** Smart Mwebembezi, Jon B. Alege, Florence Nakaggwa, Rose C. Nanyonga

**Affiliations:** Institute of Public Health and Management, Clarke International University, P.O. Box 7782, Kampala, Uganda

## Abstract

Prostate cancer accounts for 20.3% of all cancers in men in sub-Saharan Africa. Early screening among at-risk groups is challenging in Uganda, with limited data on prostate cancer screening uptake in most districts, including newly established ones. The purpose of this study was to determine factors influencing the uptake of prostate cancer screening among men aged ≥ 40 in Kazo Town Council, Kazo District, a newly created district. We used a descriptive cross-sectional study design that employed both quantitative and qualitative data collection methods. Participants were recruited through simple random sampling between November 2020 and January 2021. Structured questionnaires were used for quantitative data (*n* = 300). Statistical analyses to determine associations were carried out using inferential and chi-square tests followed by logistic regression. In-depth interviews were conducted with 10 key informants and analyzed thematically to explore a range of perceptions related to prostate cancer screening. Only 10 (3.33%; 95% CI: 0.018-0.60) respondents had ever screened for prostate cancer. Lack of privacy (*p* < 0.033), access to prostate cancer information (*p* < 0.014), and distance to health facilities (*p* < 0.001) were significantly associated with the uptake of prostate cancer screening. Marital status (OR = 7.93; 95% CI: 1.85-33.99; *p* = 0.005), positive health worker attitudes (OR = 0.002; 95% CI: 0.000-0.023, *p* < 0.001), and perceived affordability (OR = 0.001; 95% CI: 0.000-0.011, *p* < 0.001) were independently associated with uptake of prostate cancer screening. Key barriers included lack of information, access to screening centres, and fear of screening. The level of uptake of prostate cancer screening was considerably low among men aged 40 and above in the Kazo District. Targeted community interventions to improve access to prostate cancer information, screening, sensitization, and addressing perceived and actual barriers are needed in newly created districts to bolster the uptake of prostate cancer screening. This has implications for prioritizing research evaluating district resource allocation to support optimized and integrated evidence-based service delivery in primary healthcare centres, especially for specialized services in newly created districts.

## 1. Introduction

Globally, prostate cancer (PCa) remains the second most common cancer among men aged 40 years and above [1, 2]. In Africa, PCa contributes to 59,500 (16.4%) new cases per year with sub-Saharan Africa accounting for 20.3% of all cancers in men [3]. Early screening, an important tool for reducing the burden of cancer, provides opportunities for early-stage detection and triggers first-line interventions to enhance the control and survival of cancers [4, 5]. In developed countries, screening for PCa using serum prostatic specific antigen (PSA) has led to early-stage detection and reduction of PCa-specific mortality [6]. Nevertheless, this practice remains a challenge in most developing countries, leading to reduced detection rates, late diagnosis and advanced disease, poor management, and increased mortality [7].

In Uganda, PCa is a significant public health burden among men with an age-standardized incidence rate of 39.6/100,000 [8]. Another study reports higher estimates at 65.0/100,000 [3]. Various studies [1, 9] have reported a steady increase (5.2%) in the incidence of PCa annually. Katongole et al. [10] reported over 2086 new cases per year and 1,177 attributed mortalities. Although early detection through routine screening is an integral component of successful prevention and PCa therapy, the practice is hardly observed among most men aged 40 and above who are at increased risk of developing the disease. Studies [9, 11] report that 55% of men screened for PCa reported late, resulting in over 60-80% of cases succumbing to the disease. Most men are not aware of PCa screening modalities and frequently miss urinary symptoms that present with PCa; consequently, due to poor routine PCa screening, the majority (87.5%) of men in Uganda present in advanced stages of the disease [1] making attempts at successful treatment very difficult [10].

Data describing presentation and outcomes for patients with PCa in Uganda are lacking, despite the 1.77 million new cases of prostate cancers that are reported annually [1]. Early screening among at-risk groups is challenging, with limited data on PCa screening uptake in most districts, including newly established ones such as Kazo Town Council, Kazo, reflecting either a low utilization of or challenges with access to PCa screening services. Proxy data from studies done in neighboring districts provide a snapshot of the magnitude of the problem that can be inferred from the area under study [12].

A growing body of literature demonstrates that various personal and healthcare system factors influence men's decisions to screen for PCa. Documented barriers and enablers to early screening are multifactorial and encompass sociodemographic, healthcare system, and policy-related factors [13, 14]. Low uptake, for example, has been linked to low levels of education [15, 16]; attitudes, fatalistic beliefs, low-risk perception, stigma, and socioeconomic status [16–19]; marital status [20, 21]; and low levels of knowledge [18, 20].

Among health system-related factors, researchers have documented attitudes of health workers [12, 22], inadequate facilities [16], perceived affordability [18, 23, 24], and access to care and information [16, 23, 25]. Based on these studies, empirical data on how individual characteristics and health system factors are associated with PCa screening varies both in findings and recommendations. However, although not consistent in their findings, the majority of studies demonstrate that these factors have a significant association with the uptake of PCa screening, and unpacking such factors offers a golden opportunity to unearth strategies for improving men's ability to screen for PCa early. Unfortunately, research on PCa and PCa-related screening in sub-Saharan Africa is still sparse relative to the burden of the disease [9].

About 75% of Uganda's disease burden is preventable, and the Ugandan government has prioritized health promotion and disease prevention to improve the health status of the population [26]. In particular, the Ministry of Health (MoH) has reprioritized budgets to refocus on preventive interventions including community mobilization, immunization, malaria prevention and control, and NCDs among other key areas [26]. Levels of health service delivery at district levels in Uganda include services at a general hospital, Health Center Four (HCIV), and HCIII serving 500,000, 100,000, and 20,000 people, respectively. New districts in Uganda have been created to increase political participation, improve social service delivery, and ensure rigorous representation [27]. However, newly created districts require large budgets and often widen the funding gaps of local governments to create or enhance collective health service delivery [27]. For example, the average startup cost per new hospital construction, human resources, medicines, and medical equipment is estimated at UGX.31.3 billion [26]. Despite the efforts of the government to prioritize primary healthcare, newly created districts occur in a context of related and continuing challenges of shortages of the health workforce at all levels, particularly frontline workers to screen and manage NCDs [28, 29]. Effective coordination and provision of quality health services in newly created districts are likely to benefit from the availability and use of reliable data on key service needs to inform appropriate decision-making. Emerging data [27, 30] from other newly created rural districts indicate a need for building local leadership capacity and appropriate budget management and financial control to enhance collective service delivery. Kazo is a newly created district, and there is an urgent need for data for proper health sector planning, intervention development, and appropriate resource allocation especially for specialized health services. This study is aimed at identifying factors influencing PCa screening uptake among men aged > 40 in Kazo Town Council, Kazo District. Examined factors encompassed sociodemographics (age, education, religion, marital status, occupation, income, and cultural beliefs) and healthcare system elements (health workers' attitudes, privacy concerns, affordability perceptions, access to cancer care, and related information). It presents the initial dataset on PCa screening uptake factors among men aged 40 and above in this specific region.

## 2. Materials and Methods

### 2.1. Study Design

We used a descriptive cross-sectional study design that employed both quantitative and qualitative data collection methods to collect data between November 2020 and January 2021 [31]. Given that notable variance exists in the current body of evidence, we used both qualitative and quantitative approaches in this study to ensure appropriateness and nuanced representation of the study findings [31].

### 2.2. Study Setting

Kazo District is located in the western region of Uganda. It is bordered by Kiruhura District to the north, Lyantonde District to the northeast, and Sembabule District to the east. Additionally, it shares its borders with Isingiro District to the southwest and Mbarara District to the west.

The estimated distance from the capital city of Uganda, Kampala, to Kazo District is approximately 292 kilometres (181 miles). Established by the authority of parliament in July 2019 to enhance service accessibility, Kazo comprises 8 administrative units, 7 subcounties, and one town council. The district has a land area of 1551 sq. km with an estimated population of 217,600. Of these, 108,900 (50%) are male. The district headquarters are in Kazo Town Council with a recorded population of 15,900 (8000 males and 7900 females) [32].

### 2.3. Study Population

The study population comprised adult males aged 40 years residing in Kazo Town Council. Participants were selected from households without prior screening. A household survey method was adopted [33] for its ability to increase the validity and reliability of the survey.

The sample size for quantitative data was estimated using the standard formula [34] for the survey method for unknown populations as given below:

(1) $$\begin{array}{c} \text{No}=\frac{z^{2}pq}{e^{2}} \end{array}$$

where No is the sample size, *e* is the precision of the study (precision of error of 5% used), *z* is the standard deviation corresponding to a 95% confidence interval which is 1.96, *p* is the proportion of men estimated to have prostate cancer screening of 17.3% based on the Uganda Cancer Institute report in 2014, and *q* = (1 − *p*) = 1 − 0.173 = 0.8725. Thus, No = (1.96^2^∗0.173∗0.872)/0.05∗0.05). 0.9604/0.0025. *N* = 231.8. Taking into account a nonresponse rate of 30% (30.100^∗^231.8) = 69.5. *N* = 301.

A study's ideal response rate is 70% or more, anything lower risks nonresponse bias. Sensitivity in the research topic, as noted by Prince [35], can contribute to this bias. Given the study's sensitivity, we opted for a conservative 30% nonresponse rate to not compromise our sample size. In terms of qualitative data, 10 key informant (KI) interviews with opinion leaders and healthcare workers who have been providing PCa screening were carried out. KIs were randomly selected from one of the wards where they routinely work.

### 2.4. Sampling Procedure

The study district was purposively chosen as one of the newly established districts. The towns, seven parishes, and 18 villages within the Town Council were selected using a combination of simple random sampling and probability proportion to size. Kazo Town Council has a total of 2745 households. The process of getting to households and respondents was also accomplished through multistage simple random sampling techniques using probability proportion to size. Villages and the corresponding population sizes of the targeted respondents (males of 40 years and above) were listed from the district. After listing the villages with their corresponding population, we randomly selected the number of households in the villages using probability proportion to size. Then, a list of households was generated with the help of the chairpersons of the selected villages or the Village Health Teams, and then a starting household was selected and then rolled on to the next nearest household systematically. In the household, respondents that were found eligible were noted and written on the paper by the interviewer, then randomly sampled for the interview.

### 2.5. Data Collection and Analysis

Five trained research assistants collected the data following voluntary informed consent. A structured research questionnaire was used to collect quantitative data on demographic and health system factors. Data were checked for completeness and clarity. We used the Statistical Package for Social Sciences (SPSS) version 26 software for analysis. Both descriptive and inferential statistical methods were used to assess sociodemographic factors (age, level of education, marital status, religion, occupation, income level, and cultural practices) and healthcare system factors (attitude of health workers, privacy issues, perceived affordability, and access to cancer care and related healthcare information) associated with uptake of PCa screening. Inferential statistics and multivariate analysis using logistic regression were carried out to determine significant associations.

Based on the initial analysis of the quantitative data, a second structured interview guide was used to collect data from KIs. KIs were purposively selected. Interviews with the KIs were conducted by the principal investigator through face-to-face interaction at the health facility, in a private room to minimize interruptions, and were audio-recorded and kept secure. Interviews assessed knowledge and awareness of PCa, risk assessment (symptom knowledge and differentiation with other illnesses), challenges to PCa screening, and key recommendations to improve PCa screening in the district. Interviews lasted 30-45 minutes and were conducted until data saturation was achieved. Data were transcribed verbatim by an experienced transcriptionist. Common themes were generated and agreed on by all researchers.

### 2.6. Ethical Approval and Consent to Participate

This study was approved by the Clarke International University Research Ethics Committee (CIUREC) and was assigned the number CLARKE-2020-36. Informed consent was obtained from the study participants before administering the questionnaire as approved by the CIUREC. We confirm that all methods were carried out in line with relevant guidelines and regulations. The study also received approval from the District Health Officer (DHO) of Kazo District.

## 3. Results

A total of 300 respondents living in Kazo Town Council enrolled in the study. The youngest respondent was 40 years old, while the oldest was 82 years (*M* = 53.41; SD = 9.50).

**Figure 1:**
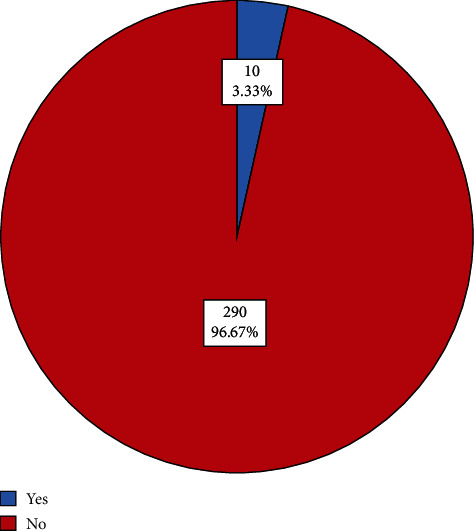
Uptake of prostate cancer screening among men aged > 40.

Nearly half (133, 44.3%) of the respondents had completed primary school education, with 66 (22%) reporting no formal education. The majority (266, 88.7%) were married, 130 (43.3%) were self-employed, and 169 (56.3%) reported monthly income levels between 30,000 UGX and 200,000 (8-56 US dollars). Over half (168, 56%) indicated that cultural practices did not influence decisions about PCa (Table 1). Only 10 (3.33%; 95%; CI: 0.018-0.60) participants had ever screened for PCa (Figure 1).

### 3.1. Factors Influencing Uptake of PCa Screening

Bivariate analysis assessing demographic factors and uptake of PCa revealed that only marital status was significantly associated with the uptake of PCa screening (*p* = 0.044) (Table 2).

In terms of health system factors, the majority (291, 97%) of respondents indicated that the attitude of health workers, lack of privacy at a health facility (299, 99.7%), and perceived affordability (291, 97%) were key barriers to uptake of PCa screening. A large proportion of the respondents (222, 75%) indicated they had heard of PCa screening. Main sources of information varied from radio (71, 23.7%) to friends (57, 19%) and doctors (44, 14.7%). In general, the hospital was the least likely (13, 4.3%) place to obtain information on PCa screening (Table 3).

Bivariate analysis of health system factors and PCa screening indicated that the attitude of health workers towards PCa screening (*p* < 0.001), lack of privacy during PCa screening (*p* < 0.033), perceived affordability of PCa screening test (*p* < 0.001), access to PCa healthcare information (*p* < 0.014), and distance to health facility (*p* < 0.001) were all significantly associated with PCa screening uptake (Table 4).

Factors independently associated with PCa screening are presented in Table 5. Being married (OR = 7.93; 95% CI: 1.85 to 33.99; *p* = 0.005) and positive attitudes from health workers (OR = 0.002; 95% CI: 0.000-0.023) were associated with increased uptake of PCa screening. Respondents who perceived PCa screening as affordable had an increased chance of uptake of PC screening (OR = 0.001; 95% CI: 0.000 to 0.011, *p* < 0.01).

### 3.2. Qualitative Results

Key informants (KI) included 2 clinical officers, 4 midwives, 3 nurses, and 1 medical doctor. Seven of the respondents (2 clinical officers, 2 nurses, 2 midwives, and 1 medical doctor) worked with the Kazo District Local Government. Of these, five worked at a primary health facility and two at the district health center. The remaining three (2 midwives and 1 nurse) were working in private health facilities. All the KIs had worked for a period of not less than three years in the health sector.

In-depth interviews explored a range of perceptions related to prostate cancer screening and assessed knowledge and awareness of PCa, risk assessment (symptom knowledge and differentiation with other illnesses), challenges to PCa screening, and key policy recommendations to improve PCa screening in the district. Key qualitative findings that aligned with the quantitative results included perceptions related to challenges to PCa screening, access to related healthcare information, and sources of information about PCa screening. Differences were noted on two questions: knowledge of PCa symptoms and at-risk category.

All respondents indicated that they had heard about PCa and believed that PCa was a common disease except for one respondent. This result corroborated with the quantitative finding where the majority (225, 75%) of respondents indicated a similar result.

In terms of PCa screening, all (10) respondents indicated that they had heard about PCa screening. Key sources of information about PCa screening were the health facility, followed by health facility doctors, while only two of the respondents indicated their source of information as relatives. This finding differed slightly from the quantitative findings where most of the respondents indicated media (especially radio), doctors, and friends as major sources of information.

KIs were also asked about their knowledge of PCa symptoms; most respondents indicated that they were knowledgeable about symptoms of PCa with the most common symptom stated as “difficult, frequent urination, blood in urine, loss of sex drive, painful sex, and infertility”. Only one respondent stated “infertility and difficult urination” as symptoms of PCa (KI1).

In terms of understanding who is at risk, KIs were asked to elaborate on who in their opinion gets PCa: six out of the ten respondents indicated that “men aged 40 to 49 years”, three indicated “men 50 to 59 years” while one stated “men 60 years and above” (KI3).

We also explored the challenges to PCa screening. The “lack of information about prostate cancer screening” coupled with “lack of community sensitization, long distance to the health facility”, and “ignorance of people” were the most reported challenges to PCa screening. One respondent indicated that “lack of testing and imaging centres was an issue,” while another stated that “people go for prostate cancer screening at a later stage while some people fear screening for prostate cancer.” Some of these results corroborated with our quantitative findings which indicated that (a) participants had multiple sources of information; (b) although they had some knowledge about PCa, the level of screening remained low due to various factors, (c) distance and lack of sensitization were key issues.

Key policy recommendations that would increase the uptake of PCa screening included “educating the community on the benefits of prostate cancer screening, creating awareness about prostate cancer, and setting up imaging centres to promote prostate cancer screening.” (KI IV).

## 4. Discussion

Our study provides the first set of data on factors influencing the uptake of PCa screening among men aged 40 and above in Kazo Town Council, a newly created district. The level of uptake of PCa screening was considerably low in this cohort. Even though the majority (225, 75%) of respondents including KIs indicated they were aware of, or had heard about PCa screening, results indicated that a large number (290, 96.67%) of the study participants had never screened for PCa. These findings are consistent with findings by Nakandi et al. [12] who reported poor knowledge, misconceptions, and poor PCa screening among adult men aged 18-71 residing in Kampala. Low uptake of PCa screening has been linked to multiple factors and barriers, such as lack of knowledge, low socioeconomic status, attitudes, and beliefs [12, 16], and increases the risk of advanced disease [36]. KI interviews supported this finding where issues such as late screening and fears about screening impacted the uptake of PCa screening in Kazo. There is a need to understand the gap that exists between perceived barriers, awareness, and uptake and what primary healthcare interventions deliver effective health promotion to support PCa screening uptake.

Among the demographic factors assessed in this study, only marital status was significantly associated with the uptake of PCa screening. Married persons were 7 times more likely to screen for PCa than those who were widowed. Studies evaluating the marital status and PCa screening uptake demonstrate that being married or being part of a family unit [20, 21, 37] is a boosting factor for PCa screening, while men who live a solitary life are less likely to go for cancer screening. Another systematic literature review by [38] demonstrated that the partner's role is the most common male-dominant enabler of health screening. This underscores the need to explore what health facilities need to do to enhance family or peer-supported interventions to bolster the uptake of PCa screening among those groups. Additionally, targeted messaging for unmarried groups and widowers (including those leading a solitary life) during PCa health promotion activities might yield better results for those groups of men and warrant some considerations.

Results of this study demonstrate that access to cancer-related health information, specifically the place (source), influences the uptake of PCa screening (*p* = 0.014). The main information source in this study was the media (radio: 71, 23.7%) and friends (57, 19%) with doctors (44, 14.7%) in third place. This was somewhat similar to a study by Wachira et al. [25] in Kenya where the majority (55.2%) of the respondents obtained information about PCa from the media while only 6.4% obtained information from a healthcare worker. Notably, the majority of respondents (77, 25%) indicated this question was not applicable. It is difficult to ascertain whether this is due to a general lack of awareness of the importance of PCa information, the relevance of information, or issues related to knowledge-seeking behaviors which were not explored in this study. Additionally, although KIs (who were all healthcare providers) perceived themselves as knowledgeable, respondents did not consider healthcare providers the first-line providers of information on PCa. Although the bivariate analysis indicated that these factors were not statistically significant in the final model, men's health literacy, in general, has been identified as a key enabler of prostate cancer screening [39]; thus, further studies are needed to demonstrate a clear picture of the barriers to information access and the mechanisms through which health promotion information can be packaged and delivered to PCa at risk-groups.

Health workers' attitudes are another factor affecting the uptake of PCa screening. Participants reported that a positive attitude among health workers, even as small as 2%, was likely to bolster uptake of PCa screening (OR = 0.002; 95% CI: 0.000-0.023). In Uganda, Salmon et al. [21] indicated that poor health workers' attitudes greatly and negatively affected PCa screening. While [16, 40, 41] reported recommendations or lack of advice/encouragement by health workers were associated with the uptake of PCa screening. PCa screening interventions need to consider the attitudes of healthcare professionals, and there is a need for the health professional to examine their attitudes to support the creation of a more receptive environment for men seeking PCa screening services.

Although income levels and uptake of PCa screening resulted in no significant findings, the perceived affordability of PCa screening test significantly influenced respondents to seek PCa screening (*p* < 0.001). Similar results have been reported by other studies including Nakandi et al. [12], Baratedi et al. [16], Bugoye et al. [18], and Kangmennaang et al. [23] The majority (69.3%) of respondents in this study reported income of <30,000 and ≤200,000/month. This may explain why affordability is a critical factor for people whose income levels may not support such an undertaking. A further analysis examining how or if income levels moderate perceived affordability was not carried out, but it may have yielded additional insight and should be an area of consideration for future studies.

The univariate analysis showed that access to privacy was another factor that was significantly (*p* = 0.033) associated with the uptake of PCa screening, though this was not significant in the regression analysis. But some studies [42] have reported that the belief that a digital rectal exam (DRE) is embarrassing deters men from pursuing PCa screening. Similar fears, including being emasculated because of the DRE, and fear of intrusion into men's personal lives were documented by Alexis and Worsley [43]. There is a degree of vulnerability associated with PCa screening, and this has implications for how screening is organized to foster a safe and enabling environment.

Recommendations by KI included “educating the community on the benefits of prostate cancer screening, creating awareness about prostate cancer, and setting up imaging centres to promote prostate cancer screening.” A well-rounded approach to establishing PCa screening services, including health promotion activities, and improvements in health system infrastructure may enhance the uptake of PCa screening in Kazo and other newly created districts in Uganda. More importantly, adopting a community health promotion approach [44] in designing men's health promotion interventions can bolster the low numbers of PCa screening in all communities.

## 5. Conclusions

The main objective of this study was to determine factors influencing the uptake of PCa screening among men aged 40 years and above in Kazo Town Council, Kazo District, a newly created district in Uganda. Data are essential for proper health sector planning, intervention development, and appropriate resource allocation. This study provides the first set of data on factors associated with the uptake of PCa screening in this district. Low levels of PCa screening underscore the need to explore mechanisms to increase uptake and minimize late risks associated with PCa. One of the important findings in this study is that marital status, specifically being married, is associated with an increased likelihood of screening for PCa, implying that companionship or some kind of peer-to-peer support is an essential consideration in PCa health promotion intervention design. It is also clear that health system factors such as the attitude of health workers play a significant role in enabling PCa screening uptake. Creating a conducive environment in which providers are perceived as knowledgeable, open, and approachable may prove a useful strategy in the development of PCa-related health promotion activities. Access and affordability are critical factors to support the uptake of PCa screening as most respondents in this sample earned income that may not support a pay-for-service alternative for PCa screening.

## 6. Recommendations

Various factors significantly impact PCa screening uptake. Targeted community interventions to improve access to prostate cancer information, screening, sensitization, and addressing perceived and actual barriers are needed in newly created districts to bolster the uptake of prostate cancer screening. Studies evaluating district resource allocation in these areas need to be prioritized to support optimized and integrated evidence-based service delivery in primary healthcare centres, especially for specialized services.

Policymakers must grasp the associations linked to PCa screening among high-risk groups, particularly men over 40, to provide information that encourages greater utilization of screening services and promotes protective behaviors against PCa.

## 7. Limitations

One of the limitations of our study is the use of self-report data which is prone to recall bias. However, we used key informant interviews to minimize bias, explore nuance, and corroborate findings. The survey result may also not be generalizable to the broader population because it predominantly includes individuals who have not undergone prostate cancer screening. As a result, the findings may not accurately represent the attitudes, behaviors, or experiences of the population at large. However, the study provides crucial data for new district leadership that may be used for comparison and decision-making.

## Figures and Tables

**Table 1:** Descriptive statistics of sociodemographic factors of study participants.

Variables	Response	Frequency (*N* = 300)	Percentage (100%)
Level of education	Never gone to school	66	22.0
	Primary	133	44.3
	Secondary	69	23.0
	Postsecondary	32	10.7

Religion	Catholic	103	34.3
	Protestants	163	54.3
	SDA	3	1.0
	Moslem	11	3.7
	Pentecostal	20	6.7

Marital status	Never married	6	2.0
	Married/cohabiting	266	88.7
	Divorced/separated	11	3.7
	Widower	17	5.7
	Total	300	100.0

Occupation	Civil servant	15	5.0
	NGO/private	15	5.0
	Farmer	104	34.7
	Casual laborer	22	7.3
	Not employed	14	4.7
	Self-employed	130	43.3

Income level	<30,000	39	13.0
	30,000-200,000	169	56.3
	200,001-500,000	68	22.7
	500,001-1,000,000	23	7.7
	>1,000,000	1	0.3

Cultural beliefs	Believe in cultural beliefs	7	2.3
	Do not believe	168	56.0
	Do not know	125	41.7

Source: *Primary Field Data 2021*.

**Table 2:** Bivariate analysis of demographic factors associated with uptake of PCa screening.

Variables	Response	Screened for prostate cancer			*χ* ^2^	*p* value
		Yes (10)	No (290)	Total		
Age	40-49 years	2 (20.0%)	116 (40.0%)	118 (39.3%)	6.073	0.123
	50-59 years	4 (40.0%)	112 (38.6%)	116 (38.7%)		
	60-69 years	3 (30.0%)	40 (13.8%)	43 (14.3%)		
	70-79 years	0 (0.0%)	17 (5.9%)	17 (5.7%)		
	80 years and above	1 (10.0%)	5 (1.7%)	6 (2.0%)		

Level of education	Nonformal	2 (20.0%)	64 (22.1%)	66 (22.0%)	3.685	0.302
	Primary	3 (30.0%)	130 (44.8%)	133 (44.3%)		
	Secondary	2 (20.0%)	67 (23.1%)	69 (23.0%)		
	Postsecondary	3 (30.0%)	29 (10.0%)	32 (10.7%)		

Marital status	Never married	0 (0.0%)	6 (2.1%)	6 (2.0%)	7.449	0.044^∗^
	Married/cohabiting	7 (70.0%)	259 (89.3%)	266 (88.7%)		
	Divorced/separated	0 (0.0%)	11 (3.8%)	11 (3.7%)		
	Widower	3 (30.0%)	14 (4.8%)	17 (5.7%)		

Religion	Catholic	4 (40.0%)	99 (34.1%)	103 (34.3%)	7.884	0.083
	Protestants	3 (30.0%)	160 (55.2%)	163 (54.3%)		
	SDA	0 (0.0%)	3 (1.0%)	3 (1.0%)		
	Moslem	2 (20.0%)	9 (3.1%)	11 (3.7%)		
	Pentecostal	1 (10.0%)	19 (6.6%)	20 (6.7%)		

Occupation	Civil servant	2 (20.0%)	13 (4.5%)	15 (5.0%)	5.752	0.215
	NGO/private	0 (0.0%)	15 (5.2%)	15 (5.0%)		
	Farmer	4 (40.0%)	100 (34.5%)	104 (34.7%)		
	Casual laborer	0 (0.0%)	22 (7.6%)	22 (7.3%)		
	Not employed	1 (10.0%)	13 (4.5%)	14 (4.7%)		
	Self-employed	3 (30.0%)	127 (43.8%)	130 (43.3%)		

Monthly income	<30,000	2 (20.0%)	37 (12.8%)	39 (13.0%)	6.421	0.179
	30,000-200,000	3 (30.0%)	166 (57.2%)	169 (56.3%)		
	200,001-500,000	3 (30.0%)	65 (22.4%)	68 (22.7%)		
	500,001-1,000,000	2 (20.0%)	21 (7.2%)	23 (7.7%)		
	>1,000,000	0 (0.0%)	1 (0.3%)	1 (0.3%)		

Cultural practices	Believe	0 (0.0%)	7 (2.4%)	7 (2.3%)	1.462	0.476
	Do not believe	4 (40.0%)	164	168 (56.0%)		
	Do not know	6 (60.0%)	119 (41.0%)	125 (41.7%)		

Source: *Primary Field Data 2021*. ^∗^Statistically significant at *p* < 0.05.

**Table 3:** Descriptive statistics of health system factors.

Variables	Response	Frequency (*N* = 300)	Percentage
Attitude of health workers	Positive	9	3.0
	Negative	291	97.0
Experience lack of privacy at the facility	Yes	1	0.3
	No	299	99.7
Affordability	Affordable	9	3.0
	Unaffordable	291	97.0
	Total	300	100.0
*Access to cancer-related healthcare information*			
Heard of prostate cancer screening	Yes	225	75.0
	No	75	25.0
Source of information	Hospital	13	4.3
	Doctors	44	14.7
	Friends	57	19.0
	Relatives	29	9.7
	Radio	71	23.7
	Television	5	1.7
	Newspaper	1	0.3
	Others	3	1.0
	Not applicable	77	25.7
Distance to health facility	4-5 km	2	0.7
	>5 km	2.3	2.3
	Do not know	291	97.0

Source: *Primary Field Data 2021.*

**Table 4:** Bivariate analysis of healthcare system factors associated with uptake of PCa Screening.

Variables	Screened for prostate cancer			*χ* ^2^	*p* value
	Yes	No	Total		
*Attitude of health workers*					
Positive	8 (80%)	1 (0.3%)	9 (3.0%)	210.771	<0.001 ^∗^
Negative	2 (20%)	289 (99.7%)	291 (97.0%)		

*Experiencing a lack of privacy*					
Yes	1 (10%)	0 (0.0%)	1 (0.35%)	29.097	0.033 ^∗^
No	9 (90%)	290 (100%)	299 (99.7%)		

*Perceived affordability of test*					
Affordable	8 (80.0%)	1 (0.3%)	9 (3.0%)	210.711	<0.001 ^∗^
Unaffordable	2 (20%)	289 (99.7%)	291 (97.0%)		

*Access to PC-related healthcare information*					
Heard of prostate cancer screening					
Yes	9 (90%)	216 (74.5%)	225 (75%)	1.241	0.239
No	1 (10%)	74 (25.5%)	75 (25%)		
Source of information					
Hospital	1 (10%)	12 (4.1%)	13 (4.3%)	17.241	0.014 ^∗^
Doctors	2 (20.0%)	42 (14.5%)	44 (14.7%)		
Friends	2 (20%)	55 (19%)	57 (19%)		
Relatives	1 (10%)	28 (9.7%)	29 (9.7%)		
Radio	1 (10%)	70 (24.1%)	71 (23.7%)		
Television	0 (0.0%)	5 (1.7%)	5 (1.7%)		
Newspaper	0 (0.0%)	1 (0.3%)	1 (0.35)		
Others	2 (20%)	1 (0.3%)	3 (1%)		
Not applicable	1 (10%)	76 (26.2%)	77 (25.7%)		

*Distance*					
4-5 km	2 (20.0%)	0 (0.0%)	nn (0.7%)	58.038	<0.001 ^∗^
>5 km	6 (60.0%)	1 (0.3%)	7 (2.3%)		
Do not know	2 (20.0%)	289 (99.7%)	291 (97.0%)		

Source: *Primary Field Data 2021*. ^∗^Statistically significant at *p* < 0.05.

**Table 5:** Model summary of factors influencing uptake of prostate cancer screening.

Variables	Wald	Df	*p* value	Odds ratio	95% CI for odds ratio	
					Lower	Upper
*Marital status*						
Married	7.773	1	0.005 ^∗^	7.929	1.85	33.987
Widower				Reference		

*Place of the source of information*	0.413	8	1.000			
Hospital	0.000	1	0.998	0.000	0.000	
Doctors	0.292	1	0.589	2.873	0.062	132.450
Pharmacy	0.056	1	0.812	1.553	0.041	58.614
Friends	0.356	1	0.551	4.840	0.027	858.110
Others				Reference		

*Health workers attitude*						
Positive	23.065	1	<0.001 ^∗^	0.002	0.000	0.023
Negative				Reference		

*Affordability of PC test*						
Positive	30.545	1	<0.001 ^∗^	0.001	0.000	0.011
Negative						

Source: *primary field data 2021*. ^∗^Statistically significant at *p* < 0.05.

## Data Availability

The data that support the findings of this study are available from Smart Mwebembezi (smartmwebs@gmail.com) upon reasonable request.

## Supplementary Materials

Supplementary Materials STROBE Statement—Checklist of items that should be included in reports of cross-sectional studies.
